# Effect of miR-27b-5p on apoptosis of human vascular endothelial cells induced by simulated microgravity

**DOI:** 10.1007/s10495-019-01580-6

**Published:** 2019-11-25

**Authors:** Yi-Kai Pan, Cheng-Fei Li, Yuan Gao, Yong-Chun Wang, Xi-Qing Sun

**Affiliations:** 1 School of Aerospace Medicine, Fourth Military Medical University, 169 Chang Le Xi Road, Xi’an, 710032 Shaanxi China; 2 Key Lab of Aerospace Medicine, Chinese Ministry of Education, Xi’an, 710032 Shaanxi China

**Keywords:** miR-27b-5p, Vascular endothelial cells, Simulated microgravity, ZHX1, Apoptosis

## Abstract

Weightlessness-induced cardiovascular dysfunction can lead to physiological and pathological consequences. It has been shown that spaceflight or simulated microgravity can alter expression profiles of some microRNAs (miRNAs). Here, we attempt to identify the role of miRNAs in human umbilical vein endothelial cells (HUVECs) apoptosis under simulated microgravity. RNA-sequencing and quantitative real-time PCR (qRT-PCR) assays were used to identify differentially expressed miRNAs in HUVECs under simulated microgravity. Then we obtained the target genes of these miRNAs through target analysis software. Moreover, GO and KEGG enrichment analysis were performed. The effects of these miRNAs on HUVECs apoptosis were evaluated by flow cytometry, Western blot and Hoechst staining. Furthermore, we obtained the target gene of miR-27b-5p by luciferase assay, qRT-PCR and Western blot. Finally, we investigated the relationship between this target gene and miR-27b-5p in HUVECs apoptosis under normal gravity or simulated microgravity. We found 29 differentially expressed miRNAs in HUVECs under simulated microgravity. Of them, the expressions of 3 miRNAs were validated by qRT-PCR. We demonstrated that miR-27b-5p affected HUVECs apoptosis by inhibiting zinc fingers and homeoboxes 1 (ZHX1). Our results reported here demonstrate for the first time that simulated microgravity can alter the expression of some miRNAs in HUVECs and miR-27b-5p may protect HUVECs from apoptosis under simulated microgravity by targeting ZHX1.

## Introduction

Weightlessness is a particular environment that can cause stress and subsequent adaption in the human body. Numerous studies have shown that real or simulated microgravity environment can lead to a high incidence of medical conditions involving cardiovascular system [1], hematological system [2], cellular immune function [3], skeletal system [4], urinary system [5], etc. These alterations induced by weightlessness are harmful to the health, working performance and safety of the astronauts. Among all these systems affected, the impaired cardiovascular control is critical issue urging us to solve. Astronauts can experience significant increases of arterial pressure and impairment of vagal baroreflex function in space [6]. Tank et al. observed orthostatic heart rate responses of astronauts before and after space flights which is diagnosed as orthostatic tachycardia [7]. Morita et al. reported that long-term exposure to microgravity environment can induce vestibulo-cardiovascular reflex impairment, which may be related to the mechanism of spaceflight-induced orthostatic intolerance [8]. Coupé et al. certified that impairment of endothelium-dependent functions caused by prolonged bed rest at the microcirculation level and endothelium should be a target for countermeasures when exposure to weightlessness [9].

The endothelium takes part in maintaining vascular homeostasis, regulating blood flow and other physiological processes [10]. Vascular endothelial cells (VECs) are a single layer of cells on the interior surfaces of vessels. The cytokines secreted by VECs play a pivotal role in mediating the balance between cholesterol and lipid [11], blood coagulation [12], signaling transduction [13], inflammatory responses [14] and so on. Because VECs are key to vascular function adjustment, endothelial deconditioning is likely to be one of important factors for the mechanism of weightlessness-induced cardiovascular dysfunction. Recent reports have showed that endothelial cells are highly sensitive to microgravity and the morphology or the function of cells is changed under this special gravity environment [15, 16]. Furthermore, previous studies have demonstrated that exposure to simulated microgravity promotes angiogenesis in HUVECs via the PI3K-AKT-eNOS signal pathway and RhoA-dependent rearrangement of the actin cytoskeleton [17, 18].

miRNAs produced by eukaryotic cells are endogenous non-coding single stranded small RNA species. They are comprised of 19–25 nucleotides and serve as master regulators of gene expression at the post-transcriptional level by pairing with the 3′untranslated region (UTR) of target mRNAs, leading to the mRNAs degradation or translation inhibition [19]. This is the main mechanism currently reported for the regulatory role of miRNAs to result in the suppression of mRNAs. It was reported that miRNAs expression profiles from different cells were significantly altered in space or simulated microgravity [20, 21]. Recently, miR-132-3p and miR-103 are identified to be up-regulated in bone loss induced by simulated microgravity [22, 23]. Moreover, RNA-Sequencing based transcriptomic profiling of neural stem cells identified many differentially expressed miRNAs between space and earth groups [24]. Zhang et al. observed that microgravity experienced in space induces transient miRNAs expression profile changes of confluent human fibroblast cells [25]. Many studies have also indicated that miRNAs are attractive candidates in cardiovascular diseases [26–29]. Although the importance of miRNAs in endothelial cells to mediate the cardiovascular functions tends to be recognized gradually [30, 31], their roles in alteration of vascular endothelial cells under simulated microgravity remain largely unknown.

The overall goal of our research is to study the effects of simulated microgravity on the expression of miRNAs and role of differentially expressed miRNAs in human vascular endothelial cells apoptosis induced by simulated microgravity. In this work, we investigated changes in the miRNAs expression profiles of HUVECs after 48 h simulated microgravity. According to the expected number of Reads per kilobase per million mapped reads (RPKM), fold change and *P* values of deep sequencing, we chose 6 miRNAs which were obviously down-regulated in MG group after 48 h simulated microgravity for further determination by PCR. Then we obtained 3 key miRNAs (miR-1268a, miR-27b-5p and miR-628-3p) which may be involved in many aspects of signal transduction between cells. After that, we reported what genes they may target to regulate the functions of HUVECs and bioinformatics analysis. More importantly, we demonstrated that miR-27b-5p might play important roles in HUVECs apoptosis under simulated microgravity via being bound to the 3′UTR of ZHX1 directly. Our results about the abnormal expression of miRNAs under 48 h simulated microgravity may provide guidelines to illustrate the molecular mechanisms of changes in human cardiovascular system during space expeditions.

## Materials and methods

### Cell culture and experimental conditions

HUVECs were purchased from American Type Culture Collection (ATCC, USA) and cultured in high-glucose Dulbecco’s modified Eagle’s medium (DMEM, Hyclone, USA) containing 10% heat-inactivated fetal bovine serum (FBS, Hyclone, USA). The cells were seeded at a density of 1 × 10^5^ cells on 2.55 × 2.15 cm coverslips in 6-well culture plates and maintained at 37 °C in a humidified atmosphere of 5% CO_2_. All experiments were conducted with confluent cultures. The cells used in the experiments were less than 6 passage numbers.

### Transfections

Mimics (miR-1268a, miR-27b-5p, and miR-628-3p), inhibitors (miR-1268a, miR-27b-5p, and miR-628-3p), their negative control oligonucleotides (mimics NC and inhibitor NC), siRNA-ZHX1, pcDNA3.1-ZHX1, siRNA-NC and pcDNA3.1-NC were all purchased from GenePharma (China). The transfections of miRNAs, siRNA and plasmid were achieved by using lipofectamine 2000 (Invitrogen, USA) according to the manufacturer’s protocol and consensus guidelines [32]. The sequences of oligonucleotides used in transfections were listed in Table 1.

**Table 1:** Sequences of oligonucleotides used in transfections

Names	Sequences (5′–3′)
miR-1268a inhibitor	CCCCCACCACCACGCCCG
miR-628-3p inhibitor	UCGACUGCCACUCUUACUAGA
miR-27b-5p inhibitor	GUUCACCAAUCAGCUAAGCUCU
miR-27b-5p mimics	AGAGCUUAGCUGAUUGGUGAAC
	UCACCAAUCAGCUAAGCUCUUU
miRNAs mimics NC	UUCUCCGAACGUGUCACGUTT
	ACGUGACACGUUCGGAGAATT
miRNAs inhibitor NC	CAGUACUUUUGUGUAGUACAA

### Clinorotation to simulate microgravity

**Fig. 1:**
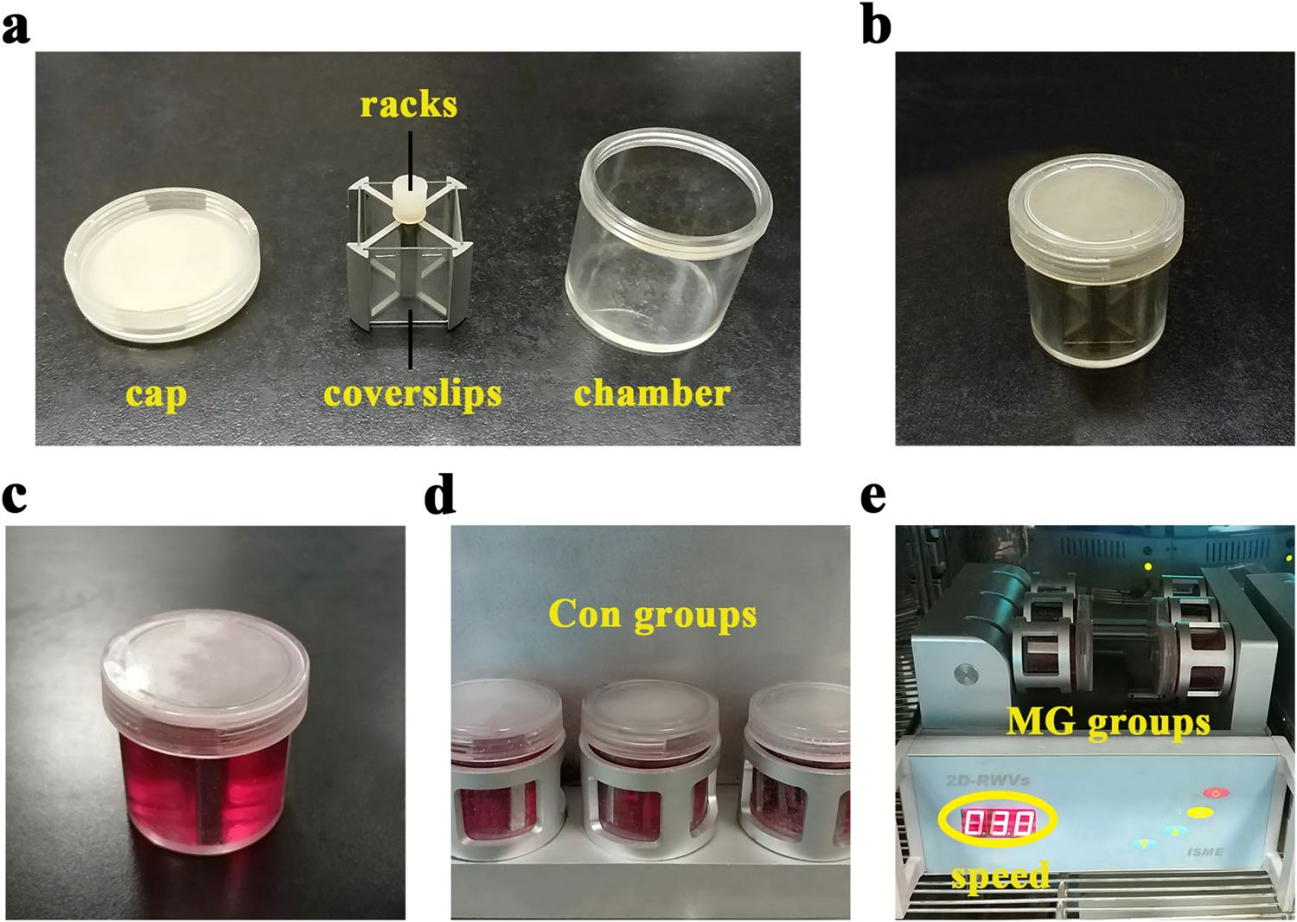
2D-clinostat and establishment of simulated microgravity system in HUVECs. **a** 2D-clinostat consists of cap, racks, coverslips and chamber. **b** The chamber without medium. **c** The chamber filled with 10% FBS, high-glucose DMEM medium. **d** The chambers of Con groups rotate around the vertical axis to act as rotation controls. **e** The chambers of MG groups rotate around the horizontal axis to avoid mechanical gravity environment. The speed of 2D-clinostat is 30 rpm

Because of the high costs of real spaceflight, most studies about the biological effects of microgravity to subjects are conducted under the condition of ground-based simulations. Among all of analog methods, the clinostat is an effective, convenient tool to simulate microgravity. A 2D-clinostat (2D-RWV, Rotating Wall Vessel, developed by China Astronaut Research and Training Center) consists of two dimensions: a vertical turntable and a horizontal turntable. The horizontal chambers rotate around the horizontal axis to avoid mechanical gravity environment. In parallel, the vertical chambers rotate around the vertical axis to act as rotation controls. The coverslips were fixed and incubated in vessel chambers completely filled with high-glucose DMEM medium containing 10% FBS after the cells grew for 24 h and adhered to the surface. The chambers were averagely divided into two groups: the clinorotation groups (MG groups) and rotation control groups (Con groups). The clinostat with chambers placed in were rotated at 30 rpm for 48 h and kept in a humidified incubator at 37 °C under 5% CO_2_ during the culture period. The structure of 2D-clinostat and the method to simulate microgravity are shown in Fig. 1.

### RNA isolation

After being cultured for 48 h in either the control or rotating condition, total RNA was extracted from HUVECs with the TRIzol reagent (Invitrogen, USA) according to the manufacturer’s instructions. RNA quality was evaluated by electrophoresis on a 1% agarose gel, and quantification was determined by using a UV-2600 spectrophotometer (UNIC, China) by the absorbance at 260 nm.

### Deep sequencing

Total RNA was sequenced with the Illumina HiSeq 2500 platform (Illumina, USA) and one small RNA library was constructed. In brief, the 10–30 nt size range of RNA was enriched by polyacrylamide gel electrophoresis to be purified and then at least 10 μg of the small RNA purification from each sample was subject to DNA sequencing according to the manufacturer’s instructions. Following ligation of the 3p and 5p adapters to the RNA in two separate subsequent steps, the small RNAs were used as templates for cDNA synthesis. Finally, the cDNA was amplified within 30 PCR cycles to produce libraries for Illumina sequencing. After removing contaminated reads, we got the clean reads of full-length small RNA sequences for further analysis.

### qRT-PCR

For miRNAs quantification, miRNA First-Strand Synthesis Kit were designed by Takara (Japan). The reactions were incubated in a thermal cycler (Applied Biosystems, USA) for 60 min at 37 °C and 5 min at 85 °C. For mRNA quantification, PrimeScript™ RT Master Mix Kit (Takara, Japan) was used to synthesize the cDNA. After reverse transcription, we used a CFX 96 instrument (Bio-Rad, USA) and SYBR Premix Ex Taq II (Takara, Japan) to carry out qRT-PCR assays according to the manufacturer’s instructions. U6 small nuclear RNA and GAPDH were performed as endogenous controls. The relative expression levels of miRNAs or mRNA between samples were calculated using the relative Ct (2^−ΔΔCt^) method and were expressed as a fold change compared with the endogenous control U6 or GAPDH. The sequences of oligonucleotides used in qRT-PCR were listed in Table 2.

**Table 2:** Sequences of oligonucleotides used in qRT-PCR

Names	Sequences (5′–3′)
miR-1268a-Forward	TATACGGGCGTGGTGGTGG
miR-27b-5p-Forward	GCTTAGCTGATTGGTGAACAA
miR-628-3p-Forward	TCTAGTAAGAGTGGCAGTCGA
U6-Forward	GGAACGATACAGAGAAGATTAGC
U6-Reverse	TGGAACGCTTCACGAATTTGCG
ZHX1-Forward	CACCTACAACAAGTTCCCTTACCC
ZHX1-Reverse	GTTTCCTTCTTGCCTCCTCTACTTC
DDIT4-Forward	CTGGCAGTTTGAGCAGCAAGA
DDIT4-Reverse	GGTTGGGTTCAGGAACAACATAATC
KANK1-Forward	AAGCAGGAGGAGGAGGGAGTTCTA
KANK1-Reverse	CCCTTGTCCTTGGAACTGCTGTA
GAPDH-Forward	AAAGGTGGAGGAGTGGGT
GAPDH-Reverse	GGGAAACTGTGGCGTGAT

### Bioinformatics analysis of sequencing data

Investigating the expression profiles of miRNAs in two groups via Illumina and making a comparison, we have harvested the differentially expressed miRNAs having large variant expression (Fold change log_2_^MG/Con^ > 2 or < − 2). In addition, the method used for normalizing expression data of miRNAs was normalized. By merging miRanda, miRDB and TargetScan, the potential target genes predicted in at least two websites were generated. To identify target genes and pathways related to biological processes of HUVECs, according to the consensus guidelines of gene expression analysis [32], we have performed a gene set enrichment analysis (GSEA, http://www.broad.mit.edu/gsea), classification by gene ontology (GO) categories (Gene Ontology, http://www.geneontology.org/), and DAVID tool (https://david.abcc.ncifcrf.gov/home.jsp). Pathway enrichment analysis was carried out based on the Kyoto Encyclopedia of Genes and Genomes Database (KEGG, http://www.genome.jp/kegg). Genes that showed statistically significance levels of *P* < 0.05 were selected.

### Flow cytometry

After 48 h transfection, cells were detached with 0.25% trypsin and washed with phosphate-buffered saline (PBS) twice. Then we isolated from 1 ml of cell suspension (1 × 10^6^ cells) by centrifugation at 1000 rpm for 5 min and mixed these samples with 5 μl Annexin V and 5 μl propidium iodide (PI) at room temperature (20–25 °C) away from light for 15 min according to the manufacturer’s recommendations. The apoptosis rates were examined by FACS Calibur flow cytometer (Becton Dickinson, USA).

### Luciferase assay

Cloning of 3′UTR of ZHX1 into pmiR-RB-REPORT™ luciferase vector, transfection and validation were conducted by Ribo Bio (China).

### Western blot assay

The expression levels of apoptosis-related proteins and ZHX1 were determined by Western blot. Total protein was extracted from HUVECs with cell lysis buffer containing phenyl-methylsulphonyl fluoride (PMSF, 1 mM), followed by centrifugation at 10,000 g at 4 °C for 15 min. The concentration of protein was determined by using the Pierce BCA Protein Assay Kit (Thermo Fisher, USA). Then the protein samples were mixed with loading buffer, boiled, electrophoresed by 12% sodium dodecyl sulphate polyacrylamide gel electrophoresis gels and transferred to the polyvinylidene difluoride membranes using the semi-dry transfer method. The membranes were blocked using 5% non-fat milk in a Tris-buffered saline-Tween 20 (TBST) solution at room temperature for 1.5 h. The primary antibodies, including Caspase-3 (1:1000), Bax (1:2000), Bcl-2 (1:2000), ZHX1 (1:2000) and GAPDH (1:2000) (Cell Signaling Technology, USA), were diluted and the membrane was then incubated at 4 °C overnight. The secondary antibody goat anti-rabbit horseradish peroxidase conjugate (Abcam, UK) were diluted 1:5000. The membrane was then incubated with the secondary antibodies at room temperature for 1 h. The protein was visualized by an ECL (enhanced chemiluminescence) detection kit (Amersham Biosciences, UK).

### Hoechst staining

A Hoechst staining kit (DiYi Biotechnology, China) was used to detect apoptosis in HUVECs according to the manufacturer’s protocol. Briefly, after washing the cells were fixed in 4% paraformaldehyde for 10 min at room temperature. Then the cells were stained with Hoechst 33342 staining solution. The fluorescence intensity of the stained cells was measured by a confocal microscopy (Zeiss Corporation, Germany).

### Statistical analysis

All numerical data are presented as mean ± SD from three independently repeated experiments. Statistical comparisons of the results were performed using Student’s *t* test or one-way ANOVA. Differences were considered statistically significant when *P* < 0.05. All statistical analysis was done with the software SPSS19.0 (SPSS Corporation, USA).

## Results

### miRNAs expression changes under simulated microgravity in HUVECs

**Fig. 2:**
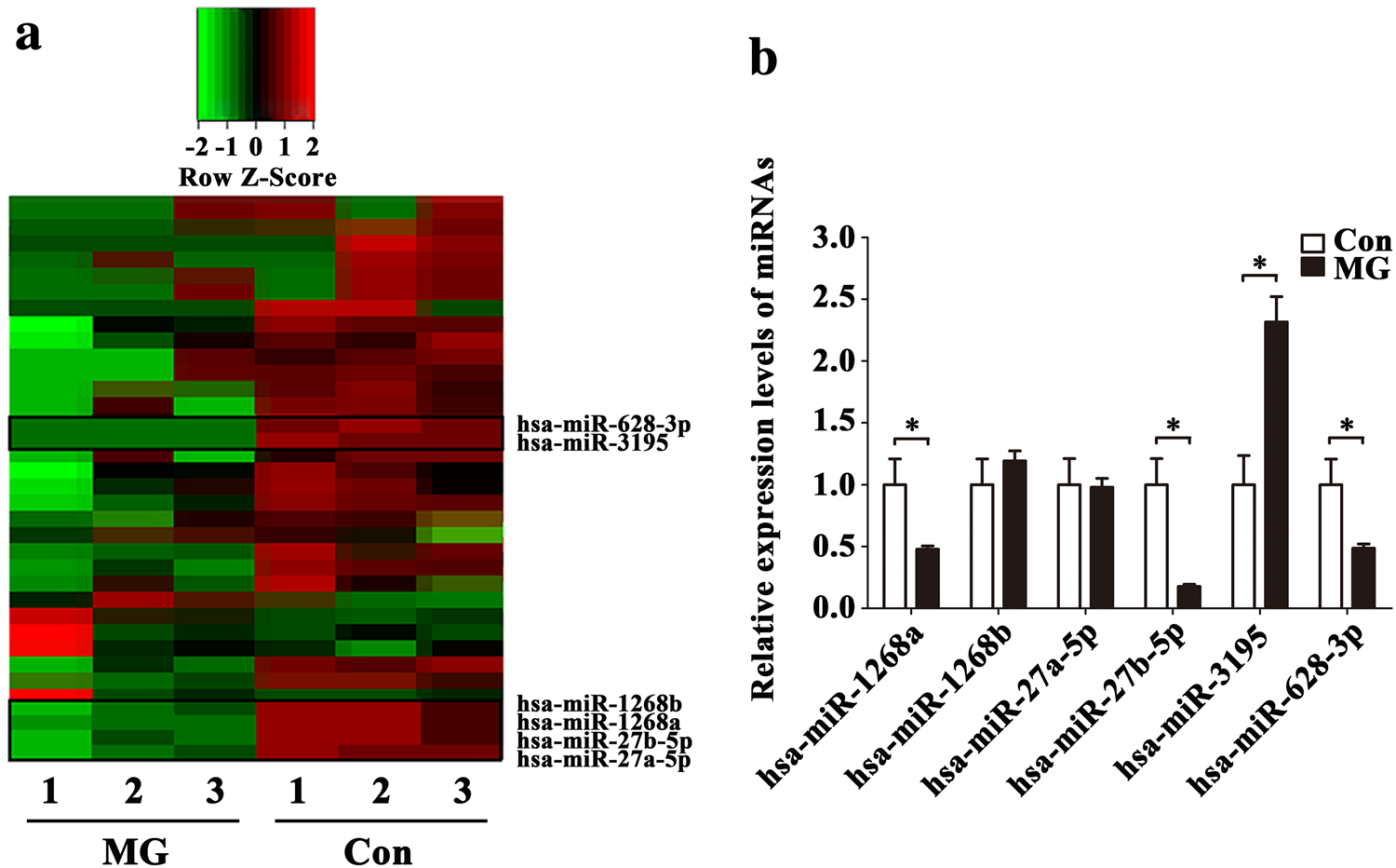
Expression changes of miRNAs in HUVECs under simulated microgravity. **a** Heat map of differentially expressed miRNAs after 48 h simulated microgravity. **b** Validation of miRNAs expression levels with qRT-PCR in HUVECs after 48 h simulated microgravity. Data are presented as mean ± SD. *N* = 3 in each group, *P < 0.05 versus Con

miRNAs were previously demonstrated to be regulated by weightlessness and involved in gene expressions. Here, we explored whether miRNAs in HUVECs could be expressed differentially after 48 h simulated microgravity. The samples were divided into two groups respectively: MG group and control group. MG group rotates around the horizontal axis to avoid mechanical gravity environment. In parallel, control group rotates around the vertical axis to act as a rotation control. Then we performed deep sequencing using two groups of samples and analyzed the data according to certain standards (fold change log_2_^MG/Con^ > 2 or < − 2). Sequencing analysis of 2588 human miRNAs showed that 15 miRNAs were down-regulated (Table 3) and 14 miRNAs were up-regulated (Table 4) after 48 h simulated microgravity in HUVECs (*P* < 0.05). We conducted the clustering for each group by hierarchical cluster, and some of the results of hierarchical cluster were shown by heatmap (Fig. 2a). In addition, according to the expression levels, fold change and *P* values, we chose 6 from down-regulated miRNAs (miR-1268a, miR-1268b, miR-27a-5p, miR-27b-5p, miR-3195 and miR-628-3p) in MG group to validate the relative expressions. The result of qRT-PCR was shown in Fig. 2b. As it can be seen, the expressions of miR-1268a, miR-27b-5p and miR-628-3p were decreased which were consistent with the results of deep sequencing, while the expression of miR-3195 was opposite. So we decided to select these 3 validated miRNAs (miR-1268a, miR-27b-5p and miR-628-3p) as candidates to conduct further experiments.

**Table 3:** Summary of significantly down-regulated miRNAs in HUVECs after 48 h simulated microgravity

Gene names	Log_2_^FC^	*P* value
hsa-miR-628-3p	− 7.2721	6.84E−11
hsa-miR-3195	− 7.0572	1.76E−09
hsa-miR-3687	− 6.4648	0.0024
hsa-miR-1257	− 6.1869	0.0031
hsa-miR-3614-5p	− 6.1104	0.0302
hsa-miR-1268a	− 6.0961	0.0039
hsa-miR-573	− 5.9028	0.0041
hsa-miR-1268b	− 5.8407	0.0043
hsa-miR-27b-5p	− 5.5699	0.0398
hsa-miR-32-3p	− 5.1827	0.0371
hsa-miR-27a-5p	− 4.8043	0.0181
hsa-miR-7974	− 4.7317	0.0203
hsa-miR-34c-5p	− 2.6132	6.12E−19
hsa-miR-23a-5p	− 2.2770	1.54E−15
hsa-miR-16-1-3p	− 2.1253	0.0434

Data are presented as mean ± SD. *N* = 3 in each group, Log_2_^FC^ < − 2, *P* < 0.05 versus Con

*FC* fold change of MG/Con ratio

**Table 4:** Summary of significantly up-regulated miRNAs in HUVECs after 48 h simulated microgravity

Gene names	Log_2_^FC^	*P* value
hsa-miR-7975	2.1346	0.0306
hsa-miR-4454	2.8561	0.0071
hsa-miR-3653-3p	3.5235	0.0456
hsa-miR-335-3p	4.2561	0.0418
hsa-miR-6894-3p	4.3405	0.0044
hsa-miR-6840-5p	4.7652	0.0038
hsa-miR-134-5p	5.1023	0.0037
hsa-miR-7114-3p	5.6554	0.0046
hsa-miR-588	5.7929	0.0024
hsa-miR-3159	5.9791	0.0018
hsa-miR-550b-3p	6.0932	0.0021
hsa-miR-486-3p	6.1408	0.0321
hsa-miR-122-5p	6.4344	0.0003
hsa-miR-3591-3p	7.1058	0.0458

Data are presented as mean ± SD. *N* = 3 in each group, Log_2_^FC^ > 2, *P* < 0.05 versus Con

*FC* fold change of MG/Con ratio

### Functional annotation for target genes of differentially expressed miRNAs

**Fig. 3:**
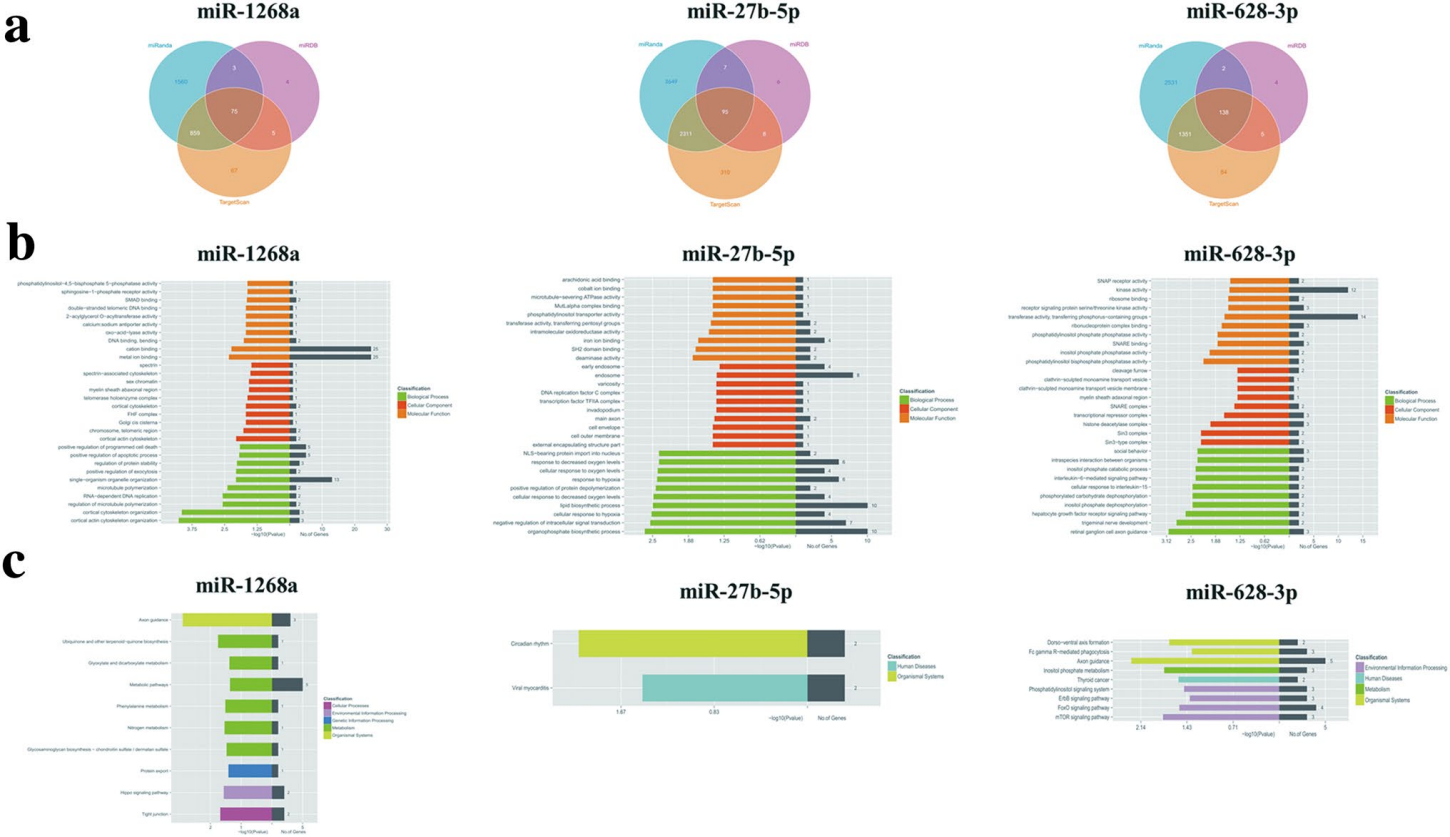
Bioinformatics analysis of target genes associated with differentially expressed miRNA validated by qRT-PCR. **a** The number of genes targeted by miR-1268a, miR-27b-5p and miR-628-3p was predicted from the data intersections of target analysis software miRanda, miRDB and TargetScan. **b** The most significant GO functions for target genes of miR-1268a, miR-27b-5p and miR-628-3p. **c** The most significant KEGG pathways for target genes of miR-1268a, miR-27b-5p and miR-628-3p

To further understand the biological function of these three differentially expressed miRNAs (miR-1268a, miR-27b-5p and miR-628-3p), we conducted bioinformatics analysis. The target genes of the above particular miRNAs were predicted by using target analysis software (miRanda, miRDB and TargetScan). Only those targets that scored in the top 5% of all predictions by at least two different programs or scored in the top 1% by any one program were included in our analysis. Using these criteria, targets for the differentially expressed miRNAs between MG and control group were identified (Fig. 3a). 75 genes targeted by miR-1268a, 95 genes targeted by miR-27b-5p and 138 genes targeted by miR-628-3p were picked out from above three miRNAs databases. In order to get credible biological functions of these intersected genes, we performed the GO (Fig. 3b) and KEGG pathway (Fig. 3c) enrichment analysis. The involvement of these target genes in different signaling network and pathways are meaningful for further validation.

### Effects of differentially expressed miRNAs on vascular endothelial cells apoptosis

**Fig. 4:**
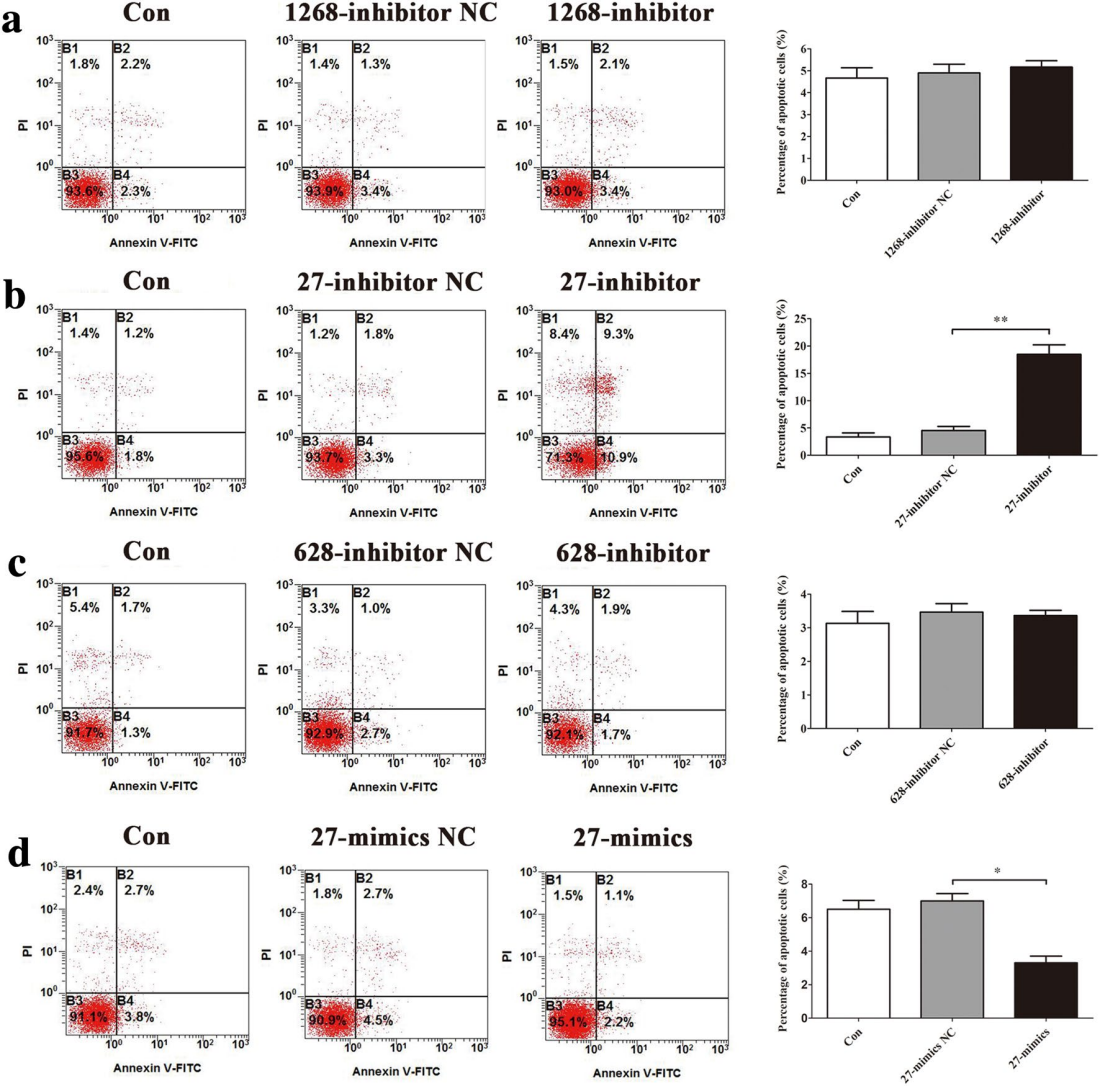
Flow cytometry analysis of differentially expressed miRNAs transfection in HUVECs apoptosis. **a** Transfection of miR-1268a inhibitor had little effect on HUVECs apoptosis. **b** miR-27b-5p inhibitor increased HUVECs apoptosis. **c** Transfection of miR-628-3p inhibitor had little effect on HUVECs apoptosis. **d** miR-27b-5p mimics decreased HUVECs apoptosis. Data are presented as mean ± SD. *N* = 3 in each group, *P < 0.05 versus 27-mimics NC, **P < 0.01 versus 27-inhibitor NC

**Fig. 5:**
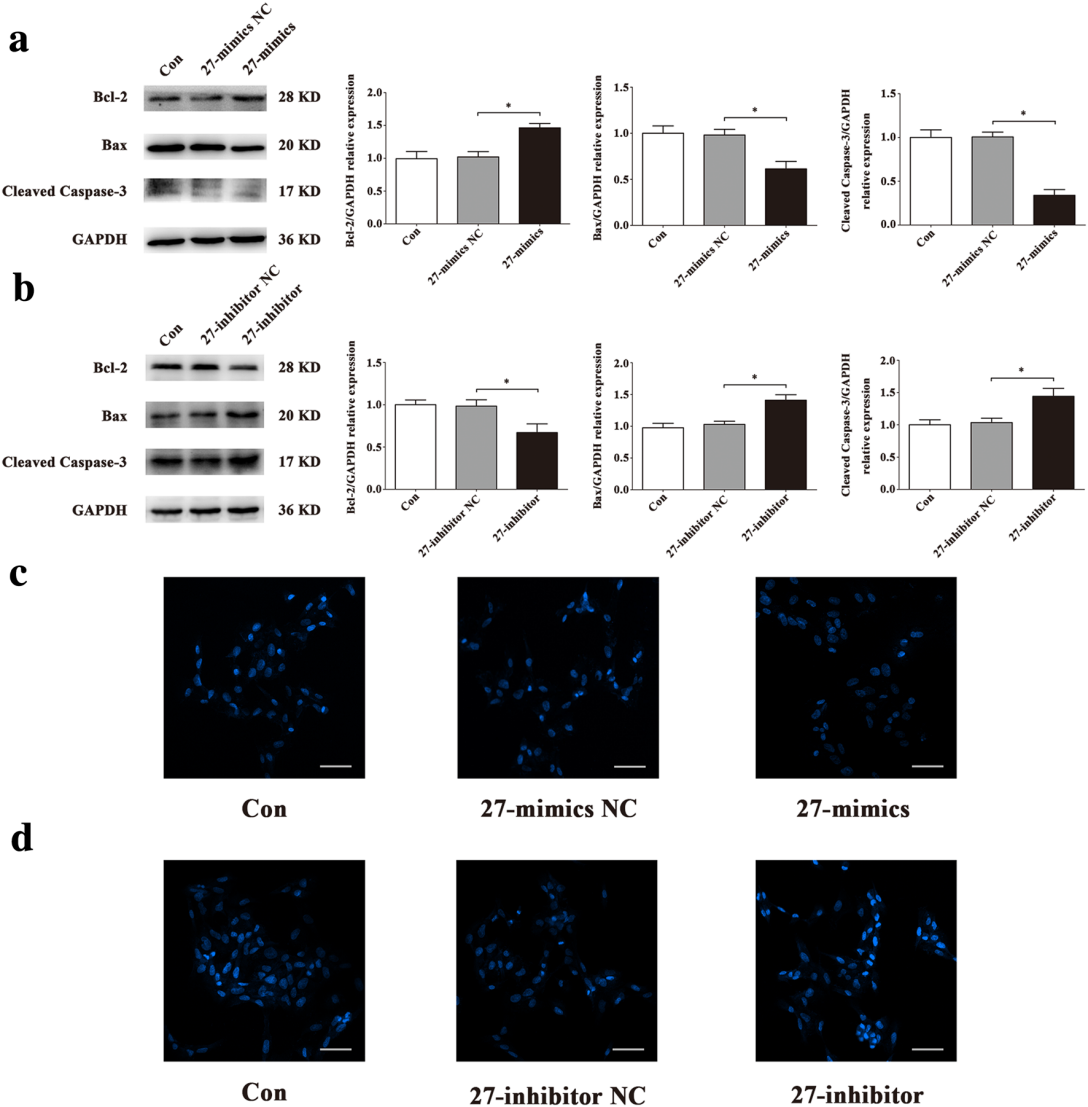
Effects of miR-27b-5p on HUVECs apoptosis. **a**, **b** Western blot analysis of apoptosis-related protein expression levels in HUVECs after transfection with 27-mimics, 27-inhibitor or their NC for 48 h. **c**, **d** Images of HUVECs stained with Hoechst 33342 after transfection with 27-mimics, 27-inhibitor or their NC for 48 h. Scale bar, 50 μm. Data are presented as mean ± SD. *N* = 3 in each group, *P < 0.05 versus 27-mimics NC or 27-inhibitor NC

**Fig. 6:**
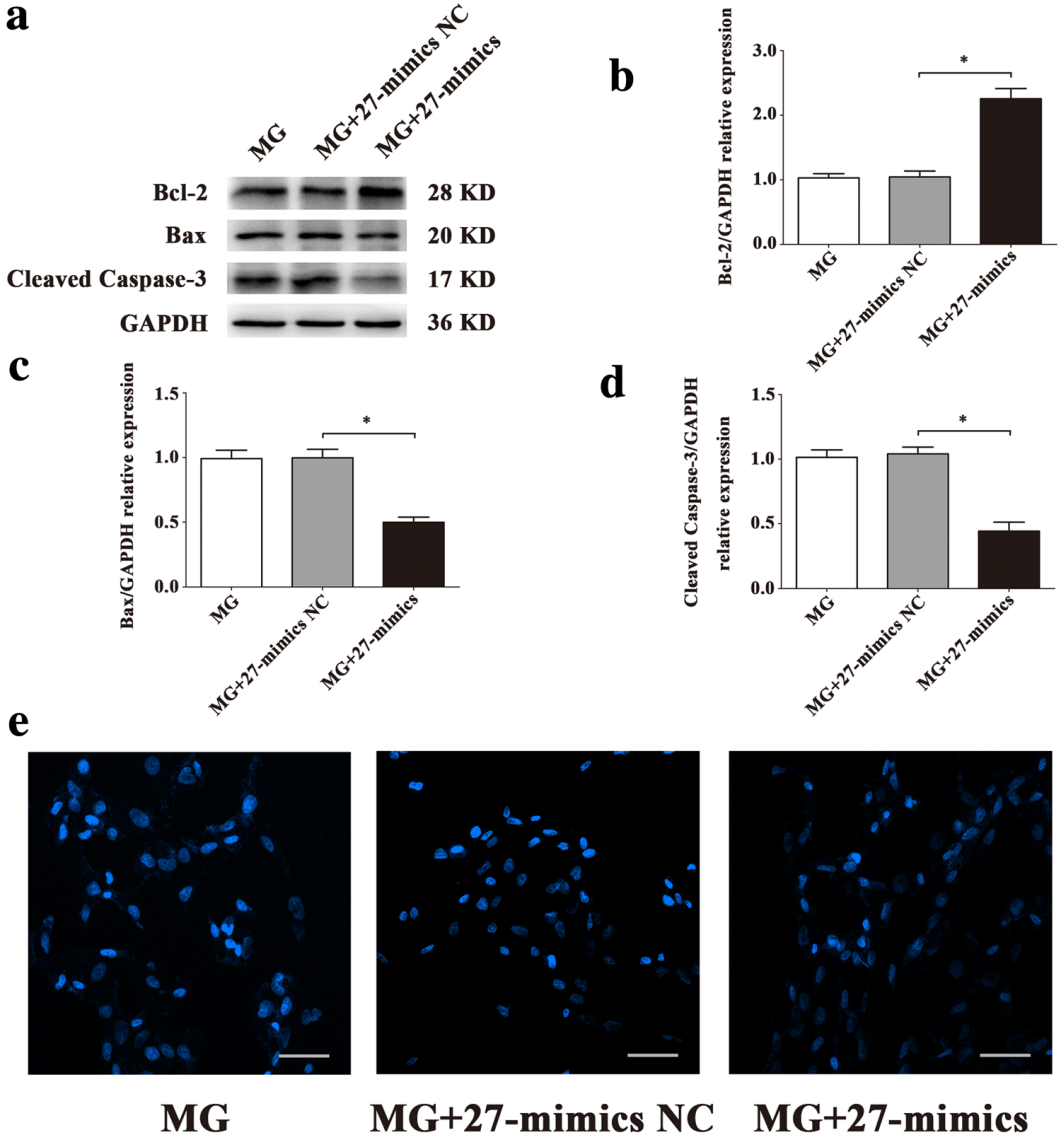
Effect of miR-27b-5p over-expression on apoptosis in HUVECs under simulated microgravity. **a–d** Western blot analysis of apoptosis-related protein expression levels in HUVECs after transfection with 27-mimics or its NC in a simulated microgravity environment for 48 h. **e** Images of HUVECs stained with Hoechst 33342 after transfection with 27-mimics or its NC in a simulated microgravity environment for 48 h. Scale bar, 50 μm. Data are presented as mean ± SD. *N* = 3 in each group, *P < 0.05 versus MG + 27-mimics NC

To explore the roles of miR-1268a, miR-27b-5p and miR-628-3p in regulating HUVECs apoptosis, we observed the apoptosis level after transfections. Flow cytometric analysis showed that miR-27b-5p inhibitor increased HUVECs apoptosis (Fig. 4b) and miR-27b-5p mimics decreased HUVECs apoptosis (Fig. 4d), compared with 27-inhibitor NC or 27-mimics NC groups, while other two miRNAs had little effects (Fig. 4a, c). Western blot assay of apoptosis-related proteins showed that miR-27b-5p mimics significantly decreased expression of Bax, Cleaved Caspase-3 and significantly increased expression of Bcl-2 in HUVECs, compared with 27-mimics NC group (Fig. 5a). In contrast, miR-27b-5p inhibitor significantly increased expression of Bax, Cleaved Caspase-3 and significantly decreased expression of Bcl-2 in HUVECs, compared with 27-inhibitor NC group (Fig. 5b). There were fewer blue apoptotic nuclei stained with Hoechst 33342 in the 27-mimics group than in the 27-mimics NC group. And there were more blue apoptotic nuclei stained with Hoechst 33342 in the 27-inhibitor group than in the 27-inhibitor NC group (Fig. 5c, d). Considering reduced expression of miR-27b-5p in simulated microgravity environment, we first transfected miR-27b-5p mimics into HUVECs to overexpress miR-27b-5p. Then we conducted the treatment of simulated microgravity for 48 h. Finally, we observed the apoptosis of HUVECs in simulated microgravity environment. Western blot results showed that the expressions of Bax and Cleaved Caspase-3 in HUVECs were significantly decreased in MG + 27-mimics NC group, compared with MG + 27-mimics NC group (*P* < 0.05), while the expression of Bcl-2 protein increased significantly (*P* < 0.05) (Fig. 6a–d). Hoechst staining showed that compared with MG + 27-mimics NC group, the number of apoptotic cells in HUVECs in MG + 27-mimics group decreased significantly (Fig. 6e). Over-expression of miR-27b-5p under simulated weightlessness can reduce the apoptosis of HUVECs. These results suggested that miR-27b-5p protects HUVECs from apoptosis under normal gravity or simulated microgravity environment.

### ZHX1 is the target gene of miR-27b-5p and promotes apoptosis in HUVECs

**Fig. 7:**
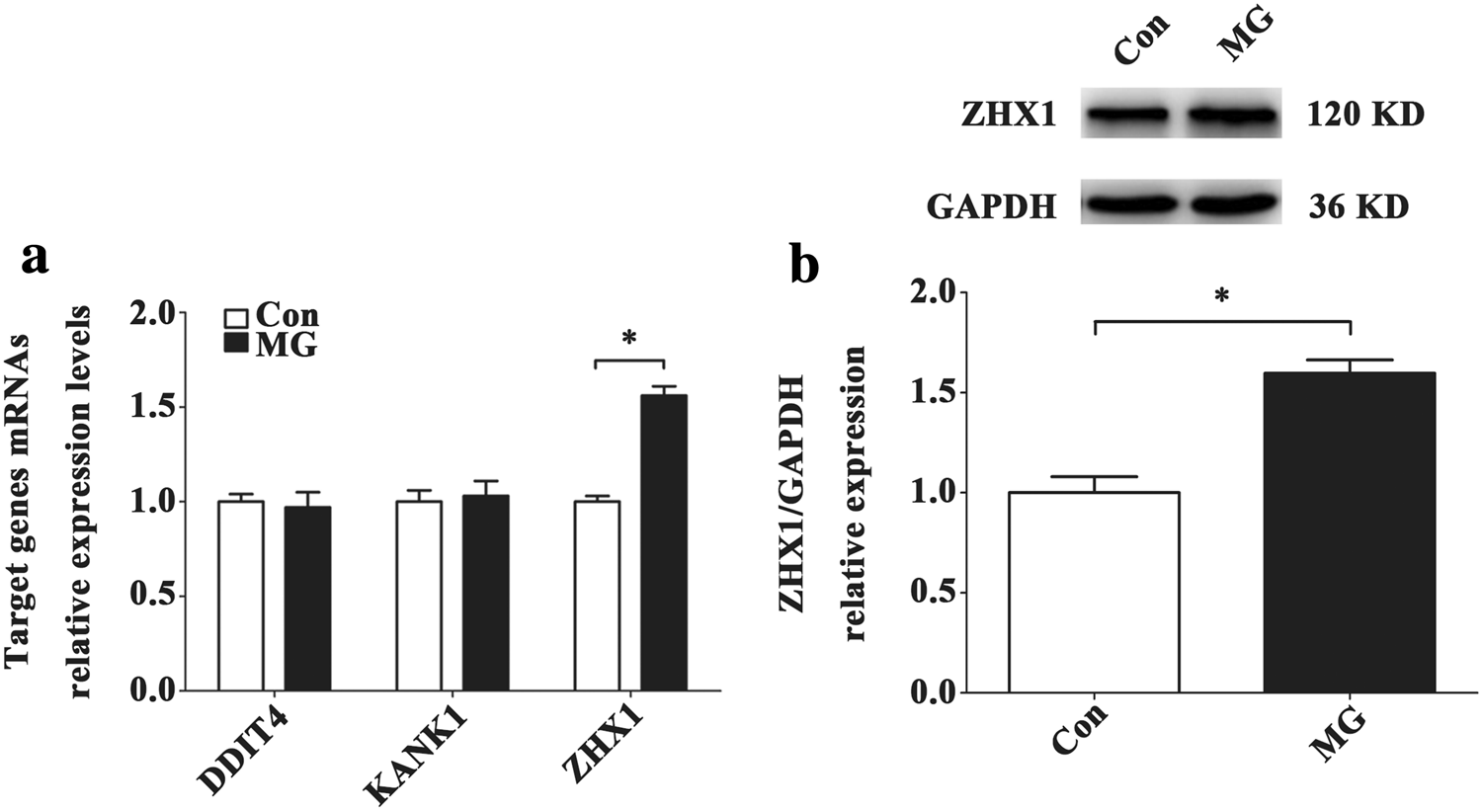
Screening and validation of target genes of miR-27b-5p in HUVECs. **a** The expression levels of potential target genes of miR-27b-5p in HUVECs after 48 h simulated microgravity were analyzed by qRT-PCR. **b** Western blot analysis of ZHX1 protein expression level in HUVECs after 48 h simulated microgravity. Data are presented as mean ± SD. *N* = 3 in each group, *P < 0.05 versus Con

**Fig. 8:**
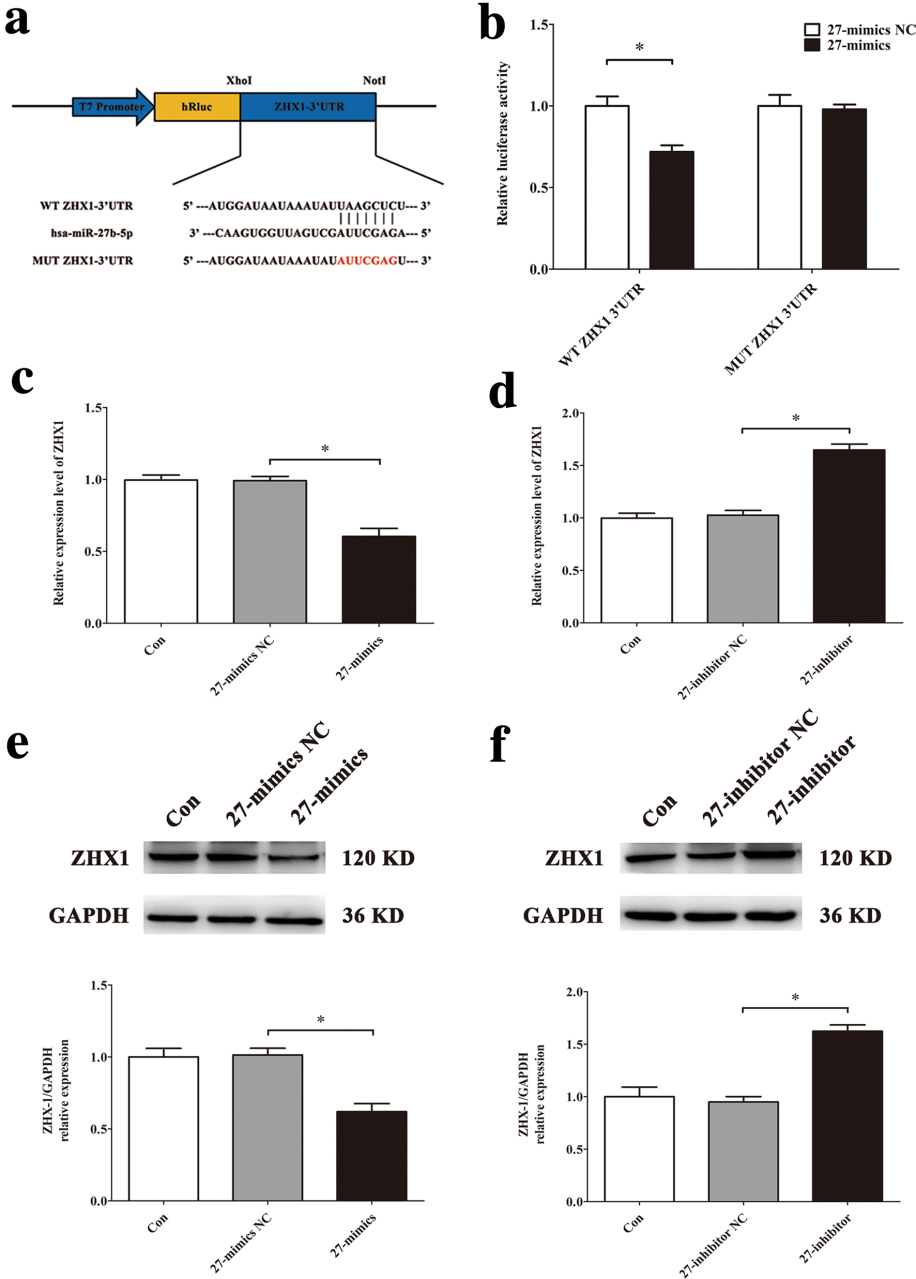
Validation of ZHX1 functioning as the target gene of miR-27b-5p in HUVECs. **a** A schematic diagram of luciferase reporters composed of pmiR-WT-ZHX1-3′UTR REPORT or pmiR-MUT-ZHX1-3′UTR REPORT. **b** The effects of miR-27b-5p mimics or its NC on the luciferase activity of pmiR-WT-ZHX1-3′UTR or pmiR-MUT-ZHX1-3′UTR REPORT in HUVECs. **c**, **d** qRT-PCR analysis of ZHX1 mRNA expression level in HUVECs after transfection with 27-mimics, 27-inhibitor or their NC for 48 h. **e**, **f** Western blot analysis of ZHX1 protein expression level in HUVECs after transfection with 27-mimics, 27-inhibitor or their NC for 48 h. Data are presented as mean ± SD. *N* = 3 in each group, *P < 0.05 versus 27-mimics NC or 27-inhibitor NC

To establish the mechanism by which miR-27b-5p affects apoptosis of HUVECs, we used the data from bioinformatics analysis (Fig. 3a) and chose the transcriptional regulator ZHX1, DDIT4 and KANK1 as the potential target gene of miR-27b-5p. And then we conducted qRT-PCR, Western blot and luciferase assay to confirm. The results of qRT-PCR showed that after 48 h of simulated microgravity, the level of ZHX1 mRNA in MG group was significantly higher than that in Con group (*P* < 0.05), while the levels of DDIT4 and KANK1 did not change significantly (Fig. 7a). In addition, we also observed the expression of ZHX1 protein after simulated microgravity. Western blot results confirmed that the expression level of ZHX1 protein in HUVECs increased significantly after 48 h of simulated microgravity (*P* < 0.05) (Fig. 7b). The luciferase reporter assay demonstrated that miR-27b-5p mimics decreased relative luciferase activity of WT ZHX1 3′UTR, but not MUT ZHX1 3′UTR luciferase reporter activity (Fig. 8b). Overexpression of miR-27b-5p decreased ZHX1 mRNA levels, while knockdown of miR-27b-5p increased ZHX1 mRNA levels (Fig. 8c, d). The protein level of ZHX1 showed the same trends (Fig. 8e, f).

**Fig. 9:**
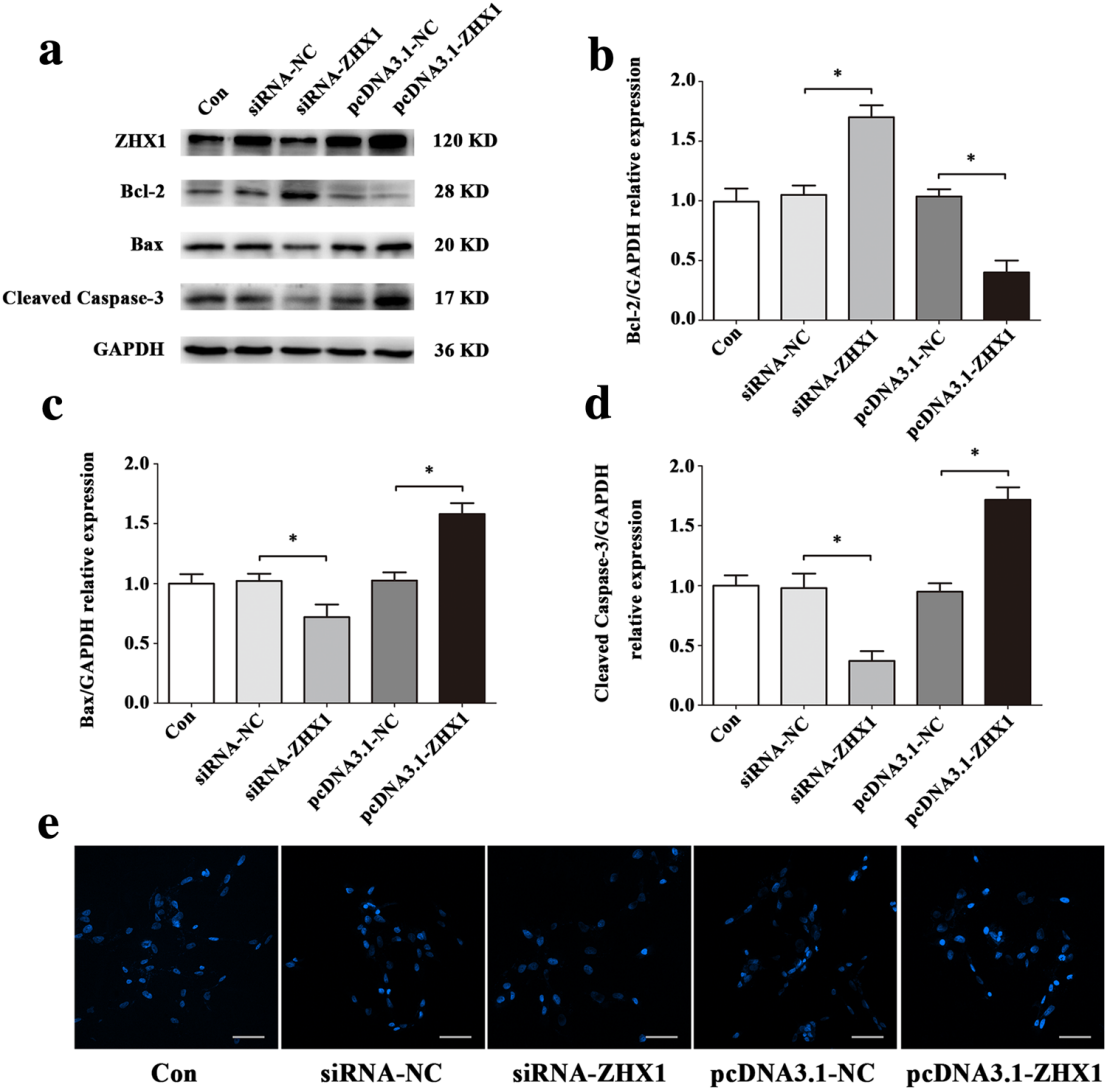
ZHX1 induces apoptosis of HUVECs. **a–d** Western blot analysis of Bcl-2, Bax and Cleaved Caspase-3 protein expression levels in HUVECs after transfection with siRNA-ZHX1, pcDNA3.1-ZHX1 or their NC for 48 h. **e** Images of HUVECs stained with Hoechst 33342 after transfection with siRNA-ZHX1, pcDNA3.1-ZHX1 or their NC for 48 h. Scale bar, 50 μm. Data are presented as mean ± SD. *N* = 3 in each group, *P < 0.05 versus siRNA-NC or pcDNA3.1-NC

**Fig. 10:**
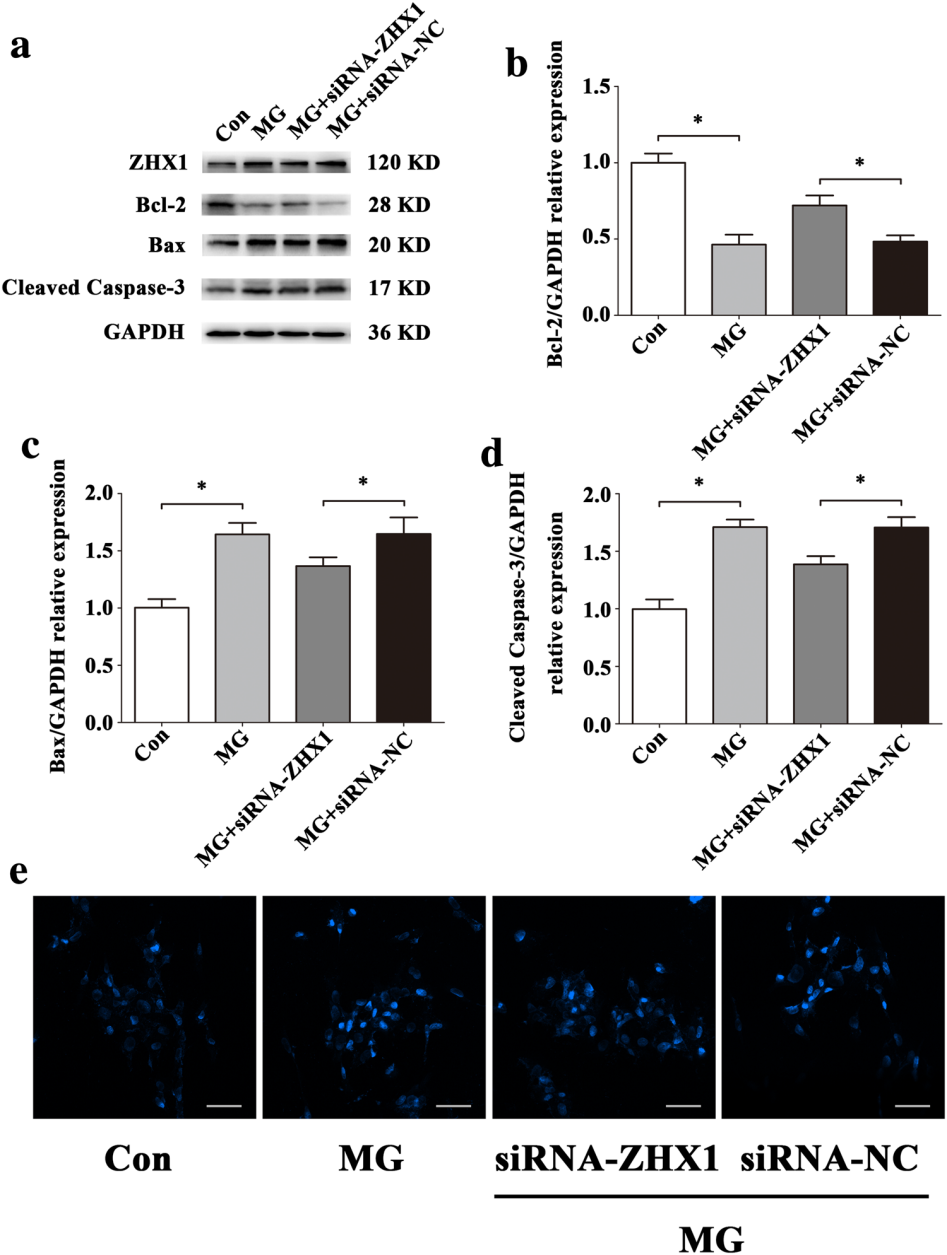
Effects of ZHX1 knockdown on apoptosis in HUVECs under simulated microgravity. **a–d** Western blot analysis of apoptosis-related protein expression levels in HUVECs after transfection with siRNA-ZHX1 or its NC under simulated microgravity environment for 48 h. **e** Images of HUVECs stained with Hoechst 33342 after transfection with siRNA-ZHX1 or its NC in a simulated microgravity environment for 48 h. Scale bar, 50 μm. Data are presented as mean ± SD. *N* = 3 in each group, *P < 0.05 versus Con or MG + siRNA-NC

To evaluate the effects of ZHX1 in apoptosis of HUVECs, we used siRNA-ZHX1 and pcDNA3.1-ZHX1 to transfect cells. The results of Western blot (Fig. 9a–d) and Hoechst staining (Fig. 9e) illustrated that upregulation of ZHX1 promoted apoptosis in HUVECs and downregulation of ZHX1 decreased apoptosis in HUVECs. In order to further verify that ZHX1 can also affect the apoptosis of HUVECs in simulated microgravity environment, we treated HUVECs transfected with ZHX1 siRNA (siRNA-ZHX1) for 48 h after simulated microgravity. Western blot and Hoechst staining were used to observe HUVECs apoptosis. Western blot results showed that the expression of Bcl-2 in HUVECs transfected with siRNA of ZHX1 was significantly increased in MG + siRNA-NC group (siRNA-ZHX1 group transfected with siRNA-ZHX group at the same time to simulate microgravity) compared with that in MG + siRNA-NC group (siRNA-ZHX group transfected with siRNA-ZHX group at the same time to simulate microgravity) (*P* < 0.05). The expression of Bax and Cleaved Caspase-3 decreased significantly (*P* < 0.05) (Fig. 10a–d). Hoechst staining showed that compared with MG + siRNA-NC group, the number of apoptotic cells in HUVECs in MG + siRNA-ZHX1 group decreased significantly (Fig. 10e). In a word, inhibiting ZHX1 expression under simulated microgravity can reduce HUVECs apoptosis.

**Fig. 11:**
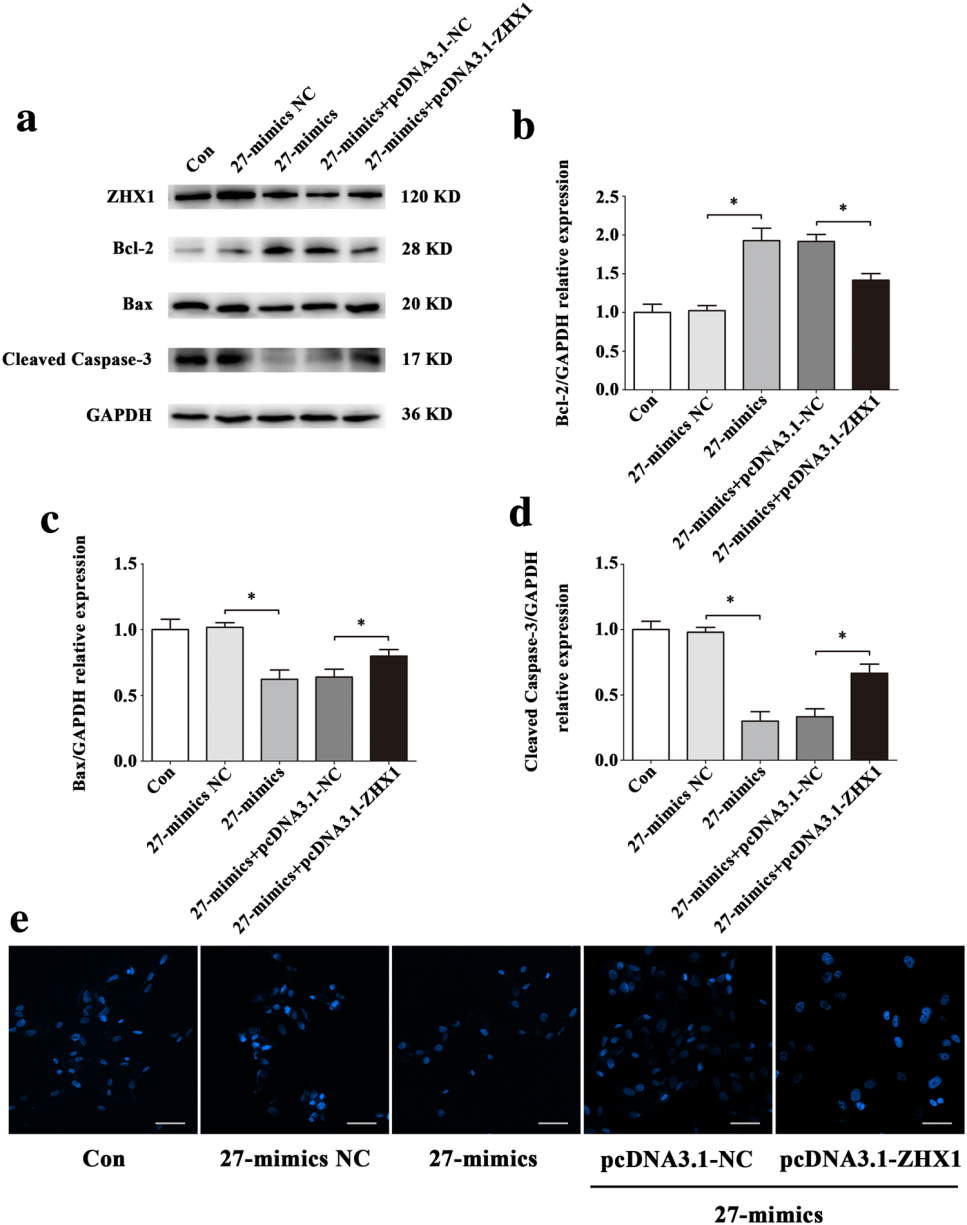
The overexpression of ZHX1 diminishes the effect of protecting HUVECs from apoptosis induced by increase of miR-27b-5p. **a–d** Western blot analysis of Bcl-2, Bax and Cleaved Caspase-3 protein expression levels in HUVECs after co-transfection with miR-27b-5p mimics and pcDNA3.1-ZHX1 or their NC for 48 h. **e** Images of HUVECs stained with Hoechst 33342 after co-transfection with miR-27b-5p mimics and pcDNA3.1-ZHX1 or their NC for 48 h. Scale bar, 50 μm. Data are presented as mean ± SD. *N* = 3 in each group, *P < 0.05 versus 27-mimics NC or 27-mimics + pcDNA3.1-NC

**Fig. 12:**
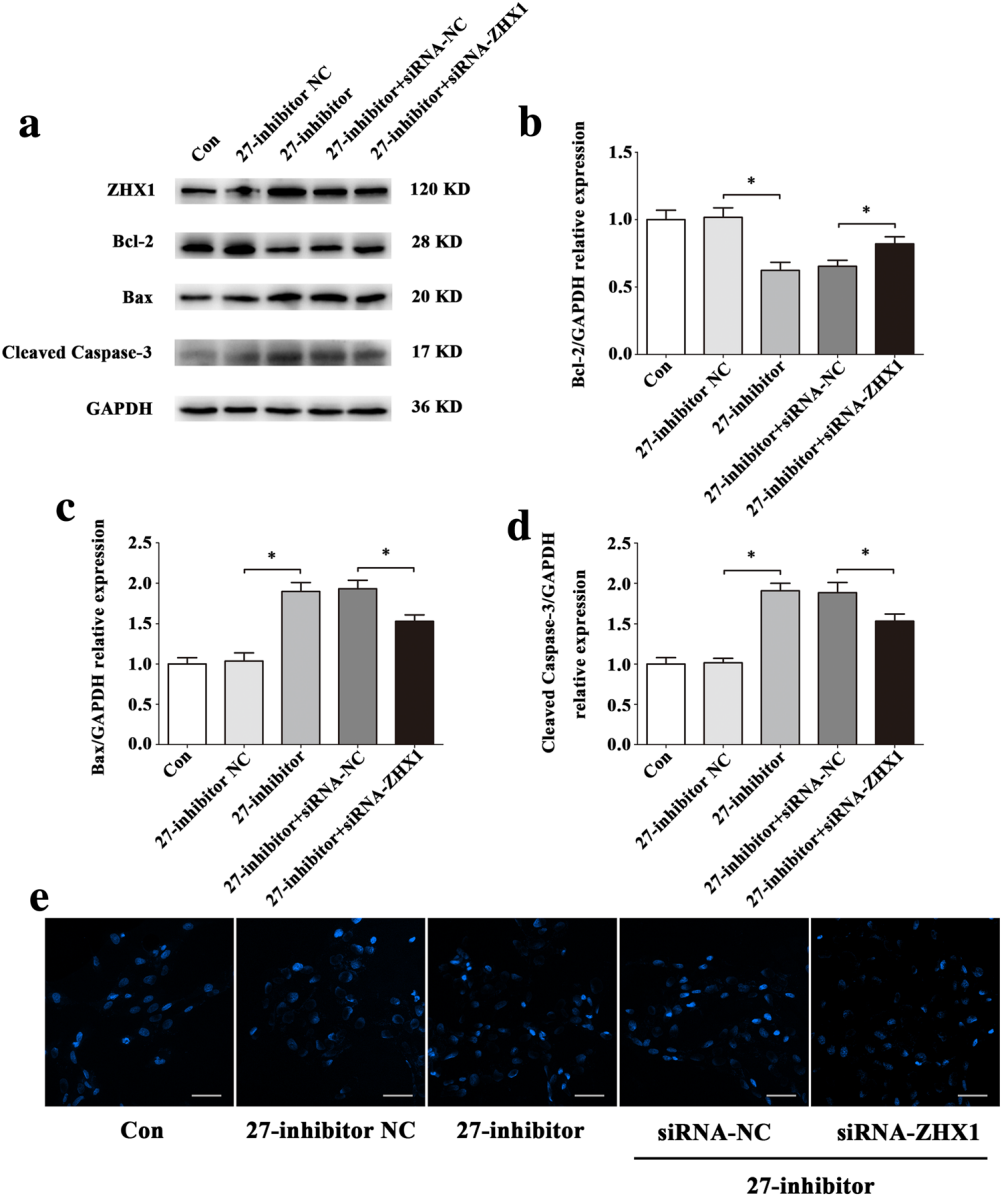
ZHX1 knock down attenuates the effect of promoting apoptosis in HUVECs induced by decrease of miR-27b-5p. **a–d** Western blot analysis of Bcl-2, Bax and Cleaved Caspase-3 protein expression levels in HUVECs after co-transfection with miR-27b-5p inhibitor and siRNA-ZHX1 or their NC for 48 h. **e** Images of HUVECs stained with Hoechst 33342 after co-transfection with miR-27b-5p inhibitor and siRNA-ZHX1 or their NC for 48 h. Scale bar, 50 μm. Data are presented as mean ± SD. *N* = 3 in each group, *P < 0.05 versus 27-inhibitor NC or 27-inhibitor + siRNA-NC

To confirm that the apoptosis of HUVECs induced by ZHX1 depends on miR-27b-5p, we conducted co-transfection assays with miR-27b-5p mimics, miR-27b-5p inhibitor, pcDNA3.1-ZHX1, siRNA-ZHX1 or their negative controls. pcDNA3.1-ZHX1 significantly inhibited the increase of Bcl-2 and decrease of Bax and Cleaved Caspase-3 induced by miR-27b-5p mimics at the protein levels (Fig. 11a–d). To evaluate the apoptotic status of HUVECs, Hoechst 33342 staining was used. As shown in Fig. 11e, more blue apoptotic nuclei were observed in the 27-mimics and pcDNA3.1-ZHX1 co-transfection group than in the 27-mimics and pcDNA3.1-NC co-transfection group. Moreover, siRNA-ZHX1 significantly attenuated the decreased Bcl-2 and increased Bax and Cleaved Caspase-3 protein levels after transfection with miR-27b-5p inhibitor (Fig. 12a–d). Compared with the 27-inhibitor and siRNA-ZHX1 co-transfection group, the number of blue apoptotic nuclei was decreased in the 27-inhibitor and siRNA-NC co-transfection group (Fig. 12e).

**Fig. 13:**
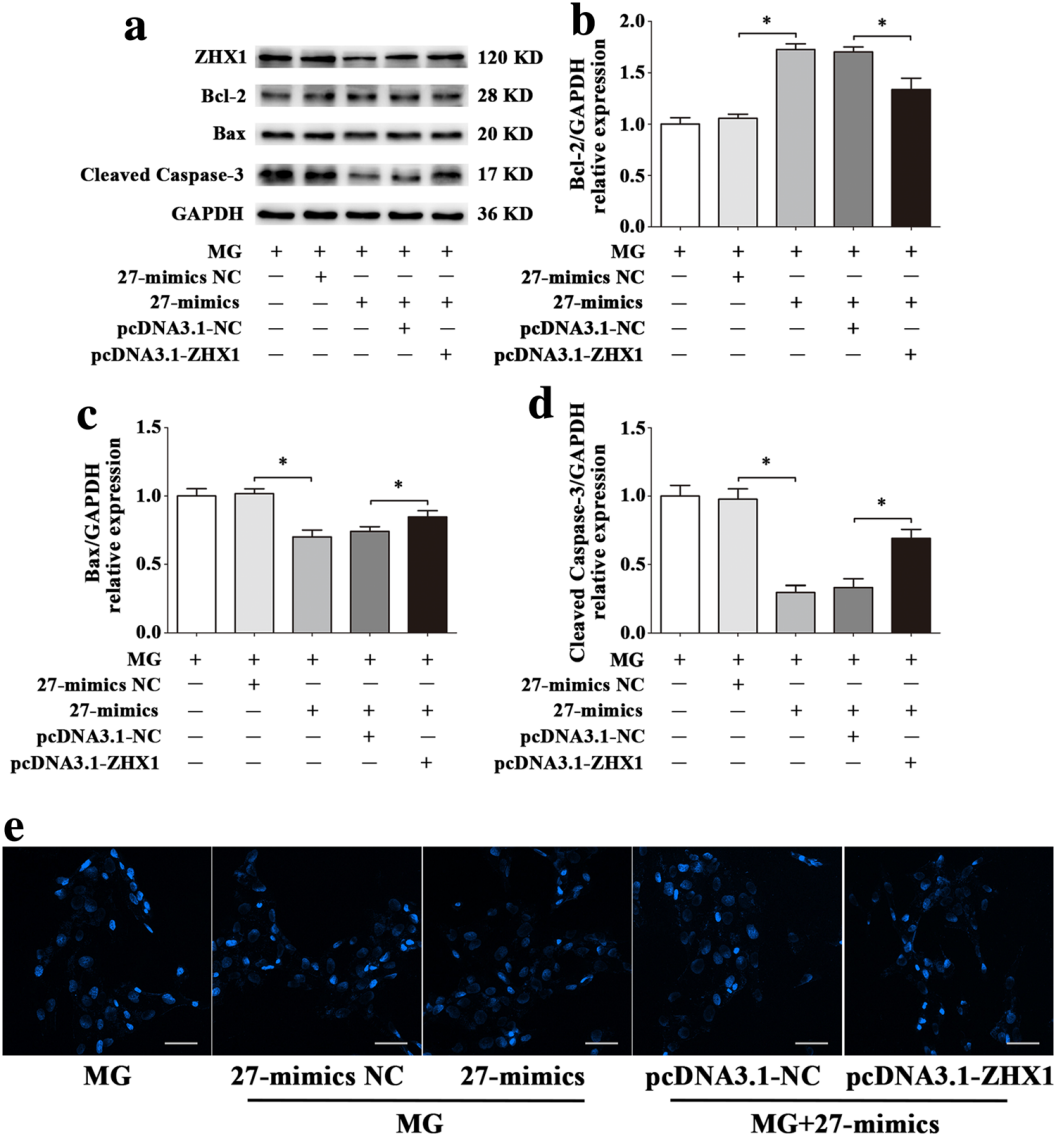
Effect of up-regulations of miR-27b-5p and ZHX1 on HUVECs apoptosis under simulated microgravity environment. **a–d** Western blot analysis of Bcl-2, Bax and Cleaved Caspase-3 protein expression levels in HUVECs after co-transfection with miR-27b-5p mimics and pcDNA3.1-ZHX1 or their NCs for 48 h simulated microgravity. **e** Images of HUVECs stained with Hoechst 33342 after co-transfection with miR-27b-5p mimics and pcDNA3.1-ZHX1 or their NCs for 48 h simulated microgravity. Scale bar, 50 μm. Data are presented as mean ± SD. *N* = 3 in each group, *P < 0.05 versus MG + 27-mimics NC or MG + 27-mimics + pcDNA3.1-NC

**Fig. 14:**
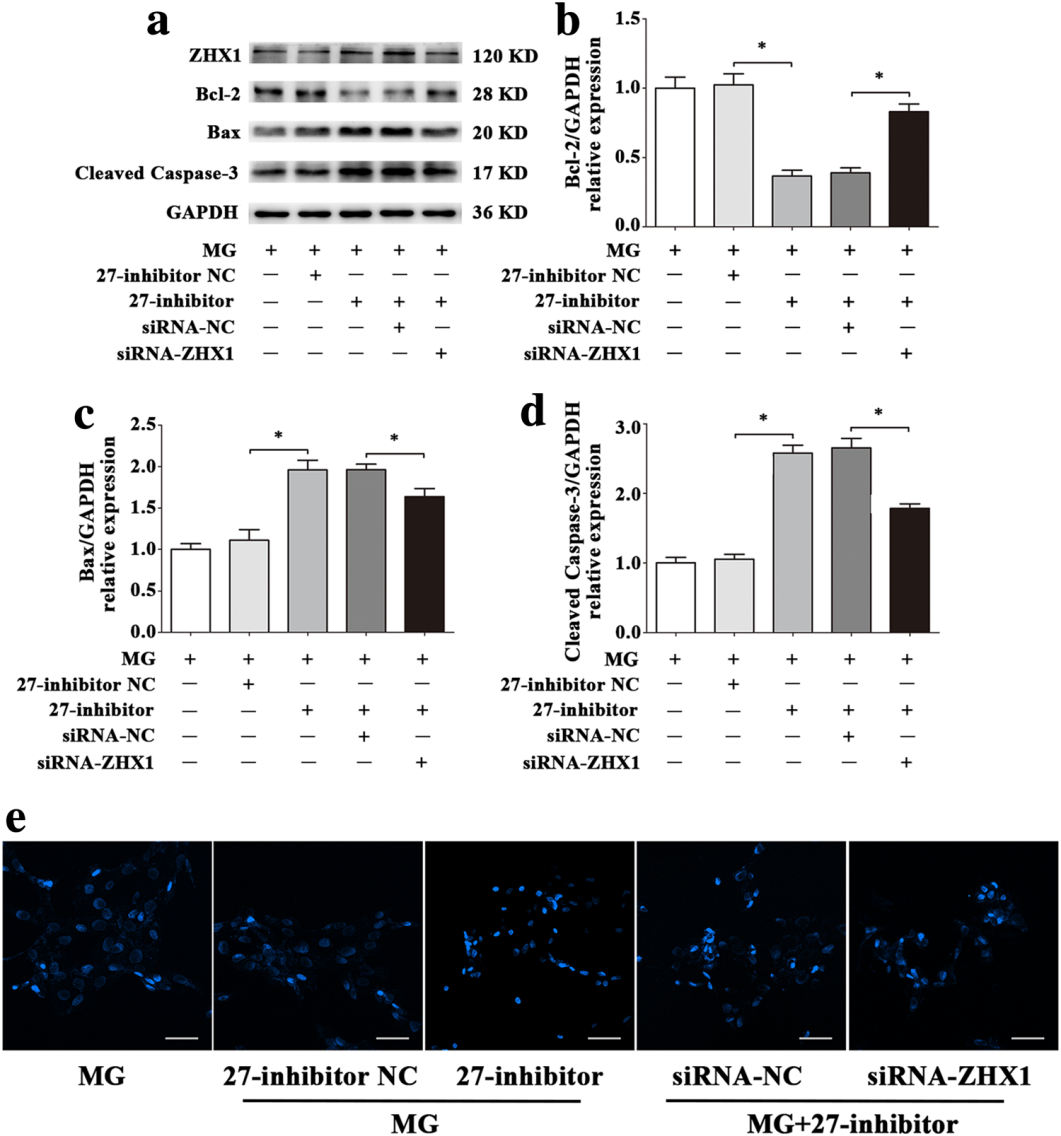
Effect of down-regulations of miR-27b-5p and ZHX1 on HUVECs apoptosis under simulated microgravity environment. **a–d** Western blot analysis of Bcl-2, Bax and Cleaved Caspase-3 protein expression levels in HUVECs after co-transfection with miR-27b-5p inhibitor and siRNA-ZHX1 or their NCs for 48 h simulated microgravity. **e** Images of HUVECs stained with Hoechst 33342 after co-transfection with miR-27b-5p inhibitor and siRNA-ZHX1 or their NCs for 48 h simulated microgravity. Scale bar, 50 μm. Data are presented as mean ± SD. *N* = 3 in each group, *P < 0.05 versus MG + 27-inhibitor NC or MG + 27-inhibitor + siRNA-NC

Because a single miRNA can regulate the expression of several target genes, it remains unknown that whether HUVECs apoptosis is regulated by miR-27b-5p via affecting the expression of ZHX1 in simulated microgravity environment. Co-transfection technique was used to up-regulate the expression of miR-27b-5p and ZHX1 in simulated microgravity environment. Western blot results showed that compared with MG + 27-mimics + pcDNA3.1-NC co-transfection group, the expression of Bcl-2 protein in MG + 27-mimics + pcDNA3.1-ZHX1 co-transfection group was significantly decreased (*P* < 0.05), while the expression of Bax and Cleaved Caspase-3 protein was significantly increased (*P* < 0.05) (Fig. 13a–d); Hoechst staining results showed that the expression of Bcl-2 protein in MG + 27-mics + pcDNA3.1-ZHX1 co-transfection group was significantly decreased (*P* < 0.05). The number of apoptotic cells in MG + 27-mimics + pcDNA3.1-ZHX1 co-transfection group was significantly higher than that in MG + 27-mimics + pcDNA3.1-ZHX1 co-transfection group, but still lower than that in MG group (Fig. 13e). Conversely, Western blot and Hoechst staining showed that apoptosis in MG + 27-inhibitor + siRNA-NC co-transfection group was significantly reduced, but still higher than that in MG group (*P* < 0.05) (Fig. 14). Combined with the above experimental results, the decrease of the expression of miR-27b-5p in simulated microgravity eliminates the inhibition of ZHX1, which leads to the increase of HUVECs apoptosis.

## Discussion

The major hypothesis of this study is that ground-based simulated microgravity would alter miRNAs expression profiles in HUVECs and differentially expressed miRNAs would take part in apoptosis of HUVECs. We used 2D-clinostat and deep sequencing technology to study the effect of simulated microgravity on HUVECs. The results indicate that some miRNAs seem to be repressed or activated during experimental conditions. In addition, we demonstrated that the role of miR-27b-5p and ZHX1 in vascular endothelial cells apoptosis. This may present a new explanation to the changes of morphology and function in HUVECs under simulated microgravity.

miR-27b-5p belongs to a subtype of miR-27b, and another subtype is miR-27b-3p, which are the mature miRNAs coming from the 5’ or 3′ ends of the same pre-miRNAs, respectively. Numerous studies have revealed the relationship between miR-27b-5p and gene expression. Chen et al. observed that miR‑27b inhibited cell proliferation and induce apoptosis in gastric cancer cells [33]. Wu et al. demonstrated that lncRNA ZEB2-AS1 promoted tumorigenesis and development of bladder cancer through down-regulating tumor-suppressive miR-27b [34]. Kim et al. identified that miR-27b-5p might be a potential biomarker for the progression of gastric cancer [35]. These discoveries above remind us that miR-27b-5p may play its role in different diseases through affecting the function of specific genes. What’s more, it has been found that miR-27b is closely related to the occurrence and development of various cardiovascular diseases. Wang et al. confirmed that miR-27b regulated by TGF-β1 could induce myocardial hypertrophy and dysfunction in mice [36]. Veliceasa et al. used coronary artery ligation to model myocardial infarction in mice. It was found that over-expression of miR-27b could increase angiogenesis and ejection fraction and reduce myocardial fibrosis [37]. Migration of monocytes regulated by MCP-1 can lead to the infiltration of inflammatory cells in the myocardium of mice with viral myocarditis. It has been found that in H9C2 myocardial cells, miR-27b can inhibit the expression of MCP-1 mRNA and protein, thereby alleviating myocardial injury [38]. Wang et al. also confirmed that the serum level of miR-27b increased significantly in elderly patients with left ventricular hypertrophy, thus providing a new idea for screening patients with left ventricular hypertrophy [39].

miR-27b is known for mediating apoptosis in cells of various origins. For example, upregulation of miR-27b reduced apoptosis in cervical cancer [40]. miR-27b increased p53-dependent cell apoptosis during Mycobacterium tuberculosis infection [41]. In addition, miR-27b attenuated apoptosis induced by transmissible gastroenteritis virus (TGEV) infection via targeting runt-related transcription factor 1 (RUNX1) in PK-15 cells [42]. Liu et al. found that miR-27b negatively regulates the proliferation of oral squamous cell carcinoma by inhibiting FZD7 and its Wnt signaling pathway [43]. What’s more, down-regulated miR-27b promotes the proliferation of retinal pigment epithelial cells by targeting Nox2 expression [44]. Thus, miR-27b is closely related to apoptosis, and can bind to different target genes in different kinds of cells to regulate the occurrence of apoptosis.

ZHX1 belongs to zinc finger structure and homeoboxes family, which also includes ZHX2 and ZHX3. ZHX1 can bind with other two family members to form heterodimers, or form homologous dimers under its own catalysis, binding locally to the nucleus to play a regulatory role in transcription. There are also many references mentioning the relationship between ZHX1 and apoptosis. Wang et al. reported that upregulation of ZHX1 led to a higher expression level of Cleaved Caspase-3 and overexpression of ZHX1 rescued the miR-199a-3p induced cell apoptosis inhibition in NCI-N87 cells [45]. Ma et al. revealed that ZHX1 could inhibit cell growth through inducing cell-cycle arrest and apoptosis in gastric cancer [46]. In addition, apoptosis of HepG2 cells was accompanied by upregulation of ZHX1 [47]. DNMT3B is one of the DNA re-methylase enzymes in mammals. Kim et al. found that ZHX1 could be used as a bridge to recruit DNMT3B to bind with other inhibitory proteins, thus promoting DNMT3B-mediated transcriptional inhibition, which to some extent explained the important inhibitory effect of ZHX1 in malignant diseases such as cancer [48]. However, it has also been reported that lncRNA DLG1-AS1 can promote the proliferation of cervical cancer cells by competitive binding with miR-107 and up-regulating the expression of ZHX1 [49]. Thus, ZHX1 has the ability to regulate cell proliferation and apoptosis which is affected by multiple factors in different types of cells. These researches provide a novel viewpoint to consider the complex relationship between miR-27b-5p and ZHX1 in HUVECs apoptosis under 48 h simulated microgravity which may be targeted for human cardiovascular dysfunction in space.

In summary, our results demonstrate that simulated microgravity environment could change the expression of miRNAs in HUVECs and select the most significant miRNAs among them for the first time. The target genes related to these miRNAs are predicted to support GO and KEGG enrichment analysis. Further study verifies that miR-27b-5p could protect vascular endothelial cells from apoptosis partially via regulating the expression of ZHX1 under simulated microgravity. Therefore, the explorations about miRNAs may improve our knowledge to thoroughly understand the regulation of gene expression in stress-related conditions like simulated microgravity.

## Abbreviations

ATCC: American type culture collection
Bax: Bcl-2 associated X protein
Bcl-2: B-cell lymphoma 2
Caspase-3: Cysteine-aspartic acid protease 3
DMEM: Dulbecco’s modifed Eagle’s medium
ECL: Enhanced chemiluminescence
FBS: Fetal bovine serum
FC: Fold change
GO: Gene ontology
GSEA: Gene set enrichment analysis
HUVECs: Human umbilical vein endothelial cells
KEGG: Kyoto encyclopedia of genes and genomes
MCP-1: Monocyte chemoattractant protein-1
MG: Microgravity
miRNAs: MicroRNAs
MUT: Mutation type
NC: Negative control
PBS: Phosphatebuffered saline
PI: Propidium iodide
PMSF: Phenyl-methylsulphonyl fluoride
qRT-PCR: Quantitative real-time PCR
RPKM: Reads per kilo-base per million mapped reads
RUNX1: Runt-related transcription factor 1
RWV: Rotating wall vessel
TBST: Tris-buffered saline-Tween 20
TGEV: Transmissible gastroenteritis virus
UTR: Untranslated region
VECs: Vascular endothelial cells
WT: Wild type
ZHX1: Zinc fingers and homeoboxes 1
